# Experimental Investigations and Effect of Nano-Powder-Mixed EDM Variables on Performance Measures of Nitinol SMA

**DOI:** 10.3390/ma15207392

**Published:** 2022-10-21

**Authors:** Rakesh Chaudhari, Yug Shah, Sakshum Khanna, Vivek K. Patel, Jay Vora, Danil Yurievich Pimenov, Khaled Giasin

**Affiliations:** 1 Department of Mechanical Engineering, School of Technology, Pandit Deendayal Energy University, Raisan, Gandhinagar 382007, India; 2 Journal of Visualized Experiments, Delhi 110016, India; 3 Department of Automated Mechanical Engineering, South Ural State University, Lenin Prosp. 76, 454080 Chelyabinsk, Russia; 4 School of Mechanical and Design Engineering, University of Portsmouth, Portsmouth PO1 3DJ, UK

**Keywords:** Nitinol, SMA, PMEDM, material removal rate, tool wear rate, surface roughness, optimization

## Abstract

In the present study, the effect of alumina (Al_2_O_3_) nano-powder was investigated for the electrical discharge machining (EDM) of a Nitinol shape memory alloy (SMA). In addition to the nano-powder concentration, other parameters of pulse-on-time (T_on_), pulse-off-time (T_off_), and current were selected for the performance measures of the material removal rate (MRR), surface roughness (SR), and tool wear rate (TWR) of Nitinol SMA. The significance of the design variables on all the output measures was analyzed through an analysis of variance (ANOVA). The regression model term has significantly impacted the developed model terms for all the selected measures. In the case of individual variables, Al_2_O_3_ powder concentration (PC), T_off_, and T_on_ had significantly impacted MRR, TWR, and SR measures, respectively. The influence of EDM variables were studied through main effect plots. The teaching–learning-based optimization (TLBO) technique was implemented to find an optimal parametric setting for attaining the desired levels of all the performance measures. Pursuant to this, the optimal parametric settings of current at 24 A, PC at 4 g/L, T_off_ at 10 µs, and T_on_ of 4 µs have shown optimal input parameters of 43.57 mg/min for MRR, 6.478 mg/min for TWR, and 3.73 µm for SR. These results from the TLBO technique were validated by performing the experiments at the optimal parametric settings of the EDM process. By considering the different user and application requirements, 40 Pareto points with unique solutions were generated. Lastly, scanning electron microscopy (SEM) performed the machined surface analysis. The authors consider this to be very beneficial in the nano-powder-mixed EDM process for appropriate manufacturing operations.

## 1. Introduction

Shape memory alloys (SMAs) are known for their superior characteristics. SMAs can remember their initial form and revert to that shape even after being subjected to bending or compression [1]. SMAs can regain their initial form even after plastic deformation by means of heating at a definite temperature or by magnetic loading. Pseudoelasticity, superelasticity, shape memory effect, and other excellent qualities of SMA make them an excellent choice for various applications [2,3]. Shape memory effect was observed in a various types of alloys [4,5]. However, the large number of applications are better suited for nickel–titanium-based SMAs. Nitinol SMA consists of an intermetal binary mixture of nickel and titanium in almost equal proportion. Nitinol has superior characteristics such as biocompatibility, superelasticity, and, more importantly, SME. Nitinol generates a protective TiO_2_ layer, which is useful for restricting the release of Ni ions in biofluid [6]. The suitability of Nitinol for biomedical applications follows from this. In addition to these features, Nitinol SMA has excellent biocompatibility and corrosion and wear resistance. For most industrial applications, the precise machining of such a sophisticated alloy becomes essential. As a result of Nitinol’s high ductility, high toughness, pseudoelasticity, and strong chemical reactivity during the process, conventional machining methods provide numerous challenges [7]. The procedures that the SMA undergoes during machining are better understood by examining the characteristics of the Nitinol alloy. Due to their austenitic matrix, alloys based on nickel harden quickly when subjected to machining. Additional machining challenges brought on by traditional machining include excessive tool wear, poor chip breakup, burr formation, and poor surface quality [8]. Due to the wide application range of Nitinol SMAs, higher geometrical precision, high cutting efficiency, and excellent surface qualities have become essential [9].

Electrical discharge machining (EDM) is a non-traditional machining technique that removes electrically conductive materials by melting and partially vaporizing the workpiece in response to the thermal energy generated by repeated sparks among the work material and tool electrode [10]. The EDM method offers a superior alternative for challenging materials to cut and is ideal for manufacturing delicate and complex profile geometries [11]. Khan et al. [12] employed a wire-EDM process to investigate the performance of Nitinol SMA. Pulse-on-time (T_on_), pulse-on-time (T_off_), wire-feed, and spark gap voltage were selected, and their effect on the machined surface was analyzed using the design of experiment (DOE) approach. Their finding revealed a recast layer thickness in the range of 7.83 to 12.13 µm near the machined surface at different parametric settings. T_on_ and T_off_ were identified as suitable machining variables. Chaudhari et al. [13] investigated the effect of the wire electrical discharge machining (WEDM) variables’ input process for a nickel–titanium-based shape memory alloy. They analyzed the material removal rate (MRR) and surface roughness (SR) performance measures. Pulse-on-time (T_on_), pulse-on-time (T_off_), and current were varied during the course of the study. The results showed that T_on_ had the largest impact for SR, whereas current contributed to enhancing the MRR response. Singh et al. [14] used the EDM method to investigate the influence of current, gap voltage, T_on_, and T_off_ on the performance measures of SR and dimensional deviation for copper-based SMA samples. SR values ranging from 4.79 µm to 8.87 µm were observed. They concluded that two factors, namely, T_on_ and current, had a significant effect on the SR measure. They also noticed some deviations in the dimensions of the workpiece from the SEM micrographs after the EDM process on Cu-based SMA.

Greater temperatures caused by high-frequency sparks result in a number of flaws such as poor surface finish, debris deposition, substantial dimensional deviations, the creation of cracks and pores, and decreased efficiency [15,16]. Finding a solution that will facilitate better MRR while minimizing SR, TWR, and other surface imperfections is very important. To improve machining, one effective method is to add nano-powders to the dielectric fluid in the proper quantity [17,18]. By enhancing spark discharge, the particle’s inclusion in the dielectric fluid lowers the breakdown strength and enhances the igniting mechanism [19]. In turn, this improves the process’s capacity for cutting. It is crucial to add suitable particles to the dielectric because too many particles might lead to machining instability and arcing issues [20]. On the other hand, excessive powder in the fluid results in a bridging effect and damage to the machined surface [21]. To improve the machining processes, researchers have used a variety of nano-powders, including Si, Cu, Al, CNT, Al_2_O_3_, Gr, Mo, and others [22]. Their performance depends on factors such as size, conductivity, and density parameters. It has been observed that the suspension of nanoparticles to the dielectric fluid substantially enhances the EDM performance [23]. The use of nanoparticles mixed with dielectric fluid improved performance measures such as cutting efficiency, SR, micro-cracks, and micro-pores [24]. In the current work, Al_2_O_3_ nano-powder, which is less expensive in comparison with commonly used nano-powders, was used. Al_2_O_3_ nano-powder has a lower density, moderate thermal conductivity, and high wear and corrosion resistance characteristics [25,26].

Kumar et al. [27] employed Al_2_O_3_ nano-powder to enhance the performance of the EDM process of Inconel 825. The MRR- and SR-obtained values were compared with those recorded from the conventional EDM process, and then they analyzed the surface morphology of the machined surface. The experimental analysis found that the largest MRR and smallest SR of 47 mg/min and 1.48 µm were attained by selecting the best possible combinations of the process parameters. This attainment has shown an improvement of 51% and 44% for SR and MRR, respectively, compared with the standard EDM technique results. MangapathiRao et al. [28] investigated the EDM process of AISI D2 steel, wherein copper tungsten electrodes and Al_2_O_3_ powder dissolved in sunflower oil were used as a substitute for EDM oil in the AISI D2 steel EDM process. This consists of selecting important process variables such as the voltage, current, T_on_, and T_off_ for TWR, MRR, and SR performance measures. The Taguchi L9 experimental design was employed to perform the trials. Analysis of variance (ANOVA) was employed to examine the influence of the process parameters on the output process response. The investigation revealed that the powder-mixed EDM (PMEDM) combined with sunflower oil and Al_2_O_3_ nanoparticles produced the least surface roughness because it absorbs heat more effectively. Increased material removal and decreased tool wear were also observed. Al_2_O_3_ nanoparticle’s abrasive effect and lowest discharge gap between particles increased the surface finish. Mohanty et al. [29] studied the performance of T_on_, current, and T_off_ variables during the PMEDM of the Al-SiC12% metal matrix composite by considering TWR, MRR, and SR as output measures. Their results have shown that the suspended nano-particles in the EDM process largely enhanced the MRR owing to the gap between the tool and the workpiece. A study conducted by Baseri and Sadeghian [30] on PMEDM using TiO_2_ nano-particles and tool rotation analyzed the output measures of TWR, MRR, and SR. As a result, it was discovered that using a rotary tool and nano-powder together could increase the effectiveness of EDM. Machining stability can be improved by increasing tool speeds by up to 200 rpm. Additionally, the maximum amount of powder that can be added before it affects the MRR is 1 g/L. Lastly, a comparison of the workpiece SEM images obtained using this approach with those obtained using traditional EDM demonstrated an improvement in surface roughness. Additionally, a magnetic field of 0.38T improves the surface roughness in this approach, raises the MRR and TWR, and makes removing depositions from the machining gap easier. Sivaprakasan et al. [31] examined the impact of graphite nano-particles for Inconel-718 for the micro-WEDM process. Voltage, capacitance, and powder concentrations were considered the primary factors affecting the output measures of kerf width, MRR, and SR. Experimental studies indicate that adding graphite nano-powder substantially improved surface morphology, reduced SR, and increased MRR. Jahan et al. [32] examined the viability of enhancing the surface finish of WC using micro-EDM through three different nano-particles of Al_2_O_3_, aluminum, and graphite. They found that the surface quality and topography were greatly enhanced owing to the larger spark gap and more consistent level of discharging. An increased spark gap and decreased dielectric breakdown strength also help improve SR while raising MRR and lowering EWR. In another study conducted by Taherkhani et al. [33], the effect of micro-sized Al_2_O_3_ particles was studied to analyze the EDM performance of the Ti6Al4V alloy. Al_2_O_3_ powder has been added to the EDM process to obtain an identical machined surface. It was discovered that using powders of a micron size during the PMEDM process can greatly improve the surface characteristics of Ti6Al4V. Thus, the studied literature clearly suggests that the use of nano-particles, specifically Al_2_O_3,_ vastly improves the performance characteristics of the EDM process.

As per the studied literature, a limited amount of work has been carried out on the performance evaluation of Nitinol SMAs through alumina nano-powder suspended in dielectric fluid. The combined effect of MRR, SR, and TWR has not been studied in depth for PMEDM, along with any investigations on surface analysis. Thus, the present study investigated alumina nano-powder-mixed EDM for the performance measures of MRR, SR, and TWR of Nitinol SMA. In addition to the nano-powder concentration, other parameters, namely, T_on_, T_off_, and current, were selected in the present study. Entire experimentations were carried out using Taguchi’s DOE approach as it offers minimum experimentations with robust parametric combinations. The significance of the design variables on all of the output measures was analyzed through analysis of variance (ANOVA). The influence of the EDM variables was studied through main effect plots. The teaching–learning-based optimization (TLBO) technique was implemented to determine the optimal parametric setting for attaining the desired levels of all the performance measures. Pareto points with unique solutions were generated by considering the need of users for specific applications. Lastly, scanning electron microscopy (SEM) was used for the machined surface analysis. The authors consider this to be very beneficial in the area of nano-powder-mixed EDM processes for appropriate manufacturing operations.

## 2. Materials and Methods

### 2.1. Alumina Nano-Powder

In the present study, the chemical reagents were used without purification. These included citric acid (C_6_H_8_O_7_), aluminum nitrate nanohydrate (Al (NO_3_)_3_·9H_2_O), Triethanolamine (N (CH_2_CH_2_OH)), and ethylene glycol (EG). All of the experiments were conducted using ultrapure water with a resistivity of 18.2 MΩ-cm. In a typical procedure, deionized water was used to dissolve aluminum nitrate nanohydrate, and a medium-speed stirrer was used to create a uniform mixture. Triethanolamine was then poured into the mixture drop by drop. After 40 min of stirring at 75 °C, citric acid was gradually added to this solution. The color of the sols changed, and, after 90 min of heating to 150 °C, the obtained sols became viscous gels. The solution was thermally heated at 1200 °C for three hours to achieve complete drying, yielding Al_2_O_3_ nano-powder. SEM (Zeiss Ultra 55 at 5 kV), X-ray diffraction (XRD) (Panalytical X’pert Pro with the source of Cu-K radiation of 0.154 nm, λ = 1.54, acceleration voltage of 45 kV and 40 mA), and Raman spectroscopy (using a laser of wavelength 532 nm) were used to characterize the synthesized Al_2_O_3_ nano-powder.

### 2.2. Experimental Details

**Figure 1:**
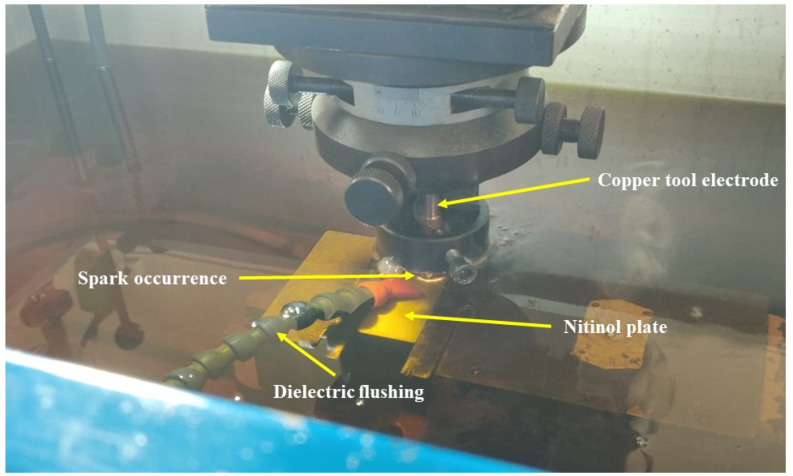
Enlarged view of the EDM experimental.

In the current work, Nitinol SMA was designated as the work material and copper as the tool electrode. Sparkonix uses die-sinking EDM for experimentations to create 10-mm diameter holes with a 2-mm depth. Figure 1 displays an enlarged view of the employed experimental setup. The designated work material contains two major elements of Ni and Ti, with an element composition of 55.8% for Ni and the remainder for Ti. The experimental setup of EDM primarily uses kerosene oil as a dielectric fluid. Alumina nano-powder was added to the dielectric tank in variable quantities to enhance the output measures. The stirrer was rotated in the dielectric tank to ensure the uniform distribution of the nano-powder. This does not permit the nano-powder from settling down in the lowermost part. In addition to the nano-powder concentration, other variables of T_on_, current, and T_off_ were selected based on studied literature and initial trials. These variables’ effects were studied based on MRR, TWR, and SR performance measures.

**Figure 2:**
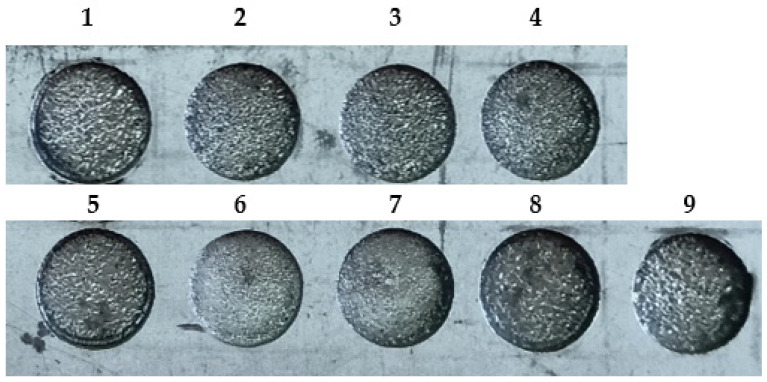
Nine Machined specimens as per the Taguch’s DOE.

Entire experimentations were carried out using Taguchi’s DOE approach as it offers minimum experimentations with robust parametric combinations [34,35]. This, in turn, saves time and cost and also provides a relationship between the design variables and performance measures [36]. Entire trials were repeated thrice to obtain superior precision and reproducibility of the results. Table 1 displays the machining conditions employed in the present work. Figure 2 shows the nine machined specimens by following Taguchi’s matrix.

The MRR and TWR were determined as per Equations (1) and (2), respectively.

(1) $$MRR=\frac{\left(W_{b}-W_{a}\right)\;}{T}$$

(2) $$TWR=\frac{\left(W_{tb}-W_{ta}\right)\;}{T}$$

where *W_b_* and *W_a_* represent the workpiece weight before and after the trials; *W_tb_* and *W_ta_* represent the tool weight before and after the trials; *T* represents the time required for the trial in minutes.

Surftest-410 was used to evaluate the SR of experimental components. SR was determined at multiple places, and the average reading was taken for examination. The cut-off length and evaluation length of 0.8 mm and 8 mm, respectively, was used to measure the R_a_ value of the machined specimens. Machined surface analysis was performed through SEM (Zeiss Ultra 55, Bangalore, India).

### 2.3. Optimization

The teaching and learning processes that take place in a classroom between a teacher and students are the foundation of the teaching–learning-based optimization (TLBO) algorithm [37]. The population of solutions is utilized in TLBO to identify the overall ideal solution. The only people in TLBO are the students in the class, and the only restrictions are the many subjects available to pupils. The grades that pupils obtain are regarded as fitness values. The highest grade a student receives in the class counts as a teacher. The teacher tries to bring the results of the other students up to their level by adjusting the mean of the marks earned by the pupils. The teacher and student phases comprise the bulk of the TLBO algorithm. The teacher phase consists of learning from the teacher, while the student phase consists of learning through interactions between the learners [38].

## 3. Results and Discussion

### 3.1. Analysis of Nano-Powder

**Figure 3:**
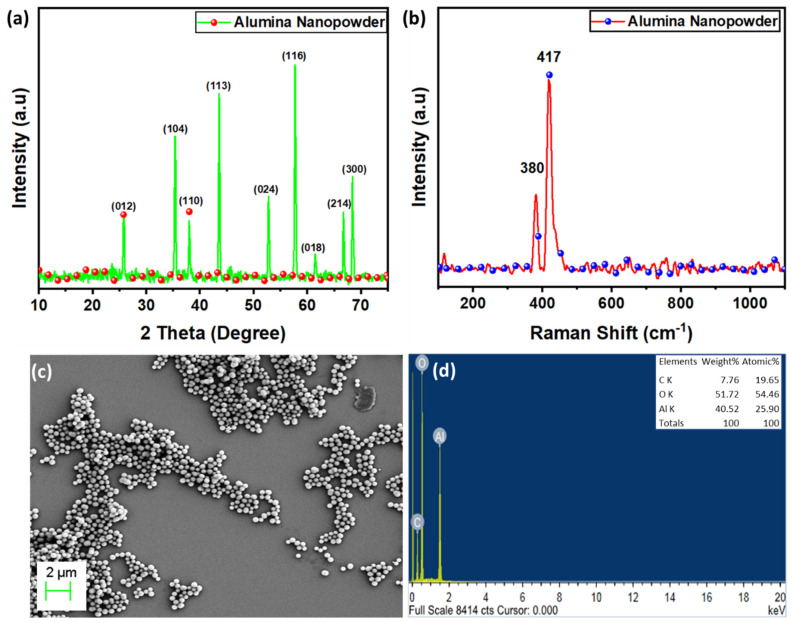
Analysis of alumina nano-powder: (**a**) X-ray Diffraction spectra, (**b**) Raman profile, and (**c**,**d**) FESEM with EDX analysis.

The prepared Al_2_O_3_ structural characteristics were examined using X-ray diffraction and Raman spectroscopy, as shown in Figure 3a,b. Peaks at 2θ~66.3°, 57.6°, 52.4°, 43.7°, 37.8°, 35.2°, and 25.3° can be attributed to the 300, 214, 116, 024, 113, 110, 104, and 012 planes (JCPDS No. 46-1212) in the diffraction profile, confirming the formation of α-Al_2_O_3_ with a hexagonal structure [39]. The average crystallite size obtained from the peaks was 27 nm using the Debye Scherrer formula. Additionally, the Raman profile (Figure 3b) displays characteristic peaks at 378 cm^−1^ and 416 cm^−1^, which were confirmed by Cava et al. [40]. Due to the absence of any additional peaks, the powders are free of impurities. Hence, the Raman spectra were found to be in agreement with the XRD findings, demonstrating the formation of α-phase Al_2_O_3_. Further, Figure 3c shows the morphology of as-produced Al_2_O_3_ nano-powder, which was examined using SEM (ULTRA 55 Carl Zeiss). The nano-powder was found to be between 100 and 150 nm in size. Energy-dispersive X-ray spectroscopy was also used to identify the elements in the as-produced Al_2_O_3_ nano-powder depicted in Figure 3d. The findings show that aluminum and oxygen are present in the synthesized material and that the only other element that was seen was carbon, which is explained by the fact that carbon is present in carbon tape.

### 3.2. Experimental Results and Regression Equations

Table 2 displays the values of the performance measures attained by following Taguchi’s matrix with the consideration of EDM variables.

All the performance measures were investigated, and a mathematical relationship through the regression equations was developed among the design variables and performance measures. The derived regression equation for MRR, TWR, and SR was represented in Equations (3)–(5), respectively.

(3) $$\mathrm{MRR}\;\left(\mathrm{mg}/\min\right)=27.54+0.399\cdot \mathrm{Current}-1.759\cdot T_{\mathrm{off}}+5.836\cdot \mathrm{PC}+0.175\cdot T_{\mathrm{on}}$$

(4) $$\mathrm{TWR}\;\left(\mathrm{mg}/\min\right)=7.087+0.0312\cdot \mathrm{Current}-0.1309\cdot T_{\mathrm{off}}-0.1274\cdot \mathrm{PC}+0.1151\cdot T_{\mathrm{on}}$$

(5) $$\mathrm{SR}\;\left(µm\right)=4.102+0.0245\cdot \mathrm{Current}-0.0950\cdot T_{\mathrm{off}}-0.1025\cdot \mathrm{PC}+0.1000\cdot T_{\mathrm{on}}$$

### 3.3. ANOVA for MRR, TWR, and SR

The statistical tool Minitab 17 was employed to study the significance of the design variables on all MRR, TWR, and SR output measures. Pursuant to this, the ANOVA technique was used to assess the acceptability of the recommended models. The confidence interval of 95% was selected to ensure the implication of the model terms. Thus, at a 95% interval, a *p*-value lower than 0.05 indicates that the respective term has a certain amount of effect on the selected output measure [41]. Table 3 represents the ANOVA results obtained for all MRR, TWR, and SR output measures. For all the selected measures, the regression model term has significantly impacted the developed model terms. This clearly shows the acceptability and adequacy of the entire model. In the case of individual variables, higher F-values/lower *p*-values have shown that PC, T_off_, and T_on_ had a significant impact on deciding MRR, TWR, and SR output measures, respectively. Apart from T_on_, all other EDM variables substantially contributed to determining the MRR response. In the case of TWR, the ANOVA table revealed that all the selected EDM variables had a substantial impact, with T_off_ being the most significant contributor, followed by T_on_, PC, and current. Similar findings were observed for SR as well, wherein entire EDM variables showed a substantial impact, with T_on_ being the most significant contributor, followed by T_off_, PC, and current. Insignificant error contributions and smaller standard deviations were recorded for performance measures. The coefficient of determinations (R^2^) was observed to be near unity with the values of 0.9766, 0.9763, and 0.9319 for MRR, TWR, and SR, respectively. This suggests the suitability of the regressions. All these obtained statistical findings revealed the acceptability and fitness of the suggested models.

### 3.4. Normal Probability Plots

**Figure 4:**
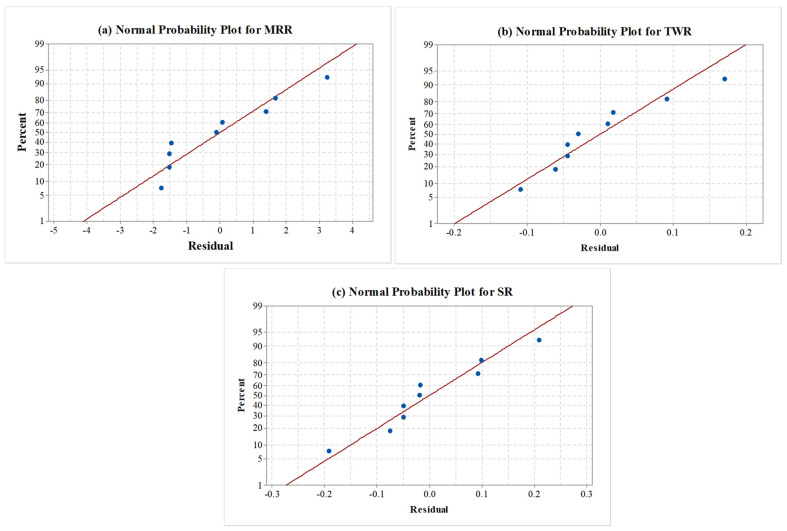
Normal probability plot for (**a**) MRR, (**b**) TWR, and (**c**) SR.

A standard probability plot was also another measure used to assess the acceptability of the ANOVA results and the adequacy of the developed regression models [42]. Thus, verification of the normal probability plot yields the validation of ANOVA results for the respective response. The normality plot confirms that the complete residuals are on a straight line. This shows that the model assumptions are correct and that they normally distributed errors [43]. Figure 4a–c illustrates that all these results were obtained for MRR, TWR, and SR responses, respectively. Thus, the obtained results of the normal probability plots for all responses imply good ANOVA results and satisfy the necessary condition for ANOVA.

### 3.5. Impact of Machining Variables on Performance Measures

This section briefly describes the influence of EDM variables on MRR, TWR, and SR performance measures through main effect plots. The statistical tool Minitab 17 was employed to plot the main effect plots for performance measures.

**Figure 5:**
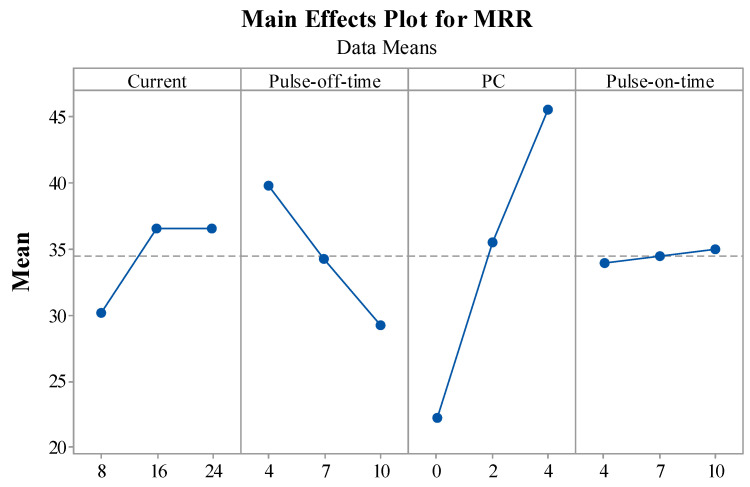
Impact of EDM variables on MRR.

Figure 5 depicts the impact of EDM variables on the output measure of MRR. EDM variables were represented on the X-axis and means of MRR for the respective input were depicted on the Y-axis. It was found that, as the current kept increasing, the MRR was also enhanced. The reason responsible for this is the increase in discharge energy owing to the current value. A rise in thermal energy melts and vaporizes the work material, enhancing the thermal energy produced by the rise in discharge energy [44]. Higher amounts of melting and vaporization cause more work material to erode at the machining zone, increasing the MRR value. For the T_off_ vs. MRR plot, a decrease in the value of MRR was observed with an increase in the value of the T_off_. An increase in the T_off_ value results in a drop in discharge energy. Spark intensity and discharge energy decrease with an increase in T_off_, and because MRR is directly proportional to discharge energy, it also decreases [45]. For the PC vs. MRR plot, it was found that, as the PC keeps increasing, the MRR also increases. A more significant amount of ions from the work material can be transferred because of the thermo-electrical properties of the nano-powder, which operate as a good conductor in the inter-electrode gap [46]. Pursuant to this, an increase in MRR can be seen when the concentration of nano-powder increases. Additionally, the quantity of sparking and thermal conductivity rises upon adding the powder to the dielectric, speeding up erosion from the work surface. The higher erosion rate thus enhances MRR even more. For T_on_ vs. MRR, it was observed that, with an increase in the value of T_on_, the value of MRR also increased. The material becomes eroded due to using a recurring spark during the machining process [47]. With an increase in T_on_, these discharges also rise. The thermal energy was increased, resulting in the workpiece melting and vaporizing. Increased melting and vaporization led to higher erosion from the work materials and, thus, to an increase in MRR. Ideal levels for obtaining a higher MRR include setting current, PC, and T_on_ at higher levels and T_off_ at a lower level.

**Figure 6:**
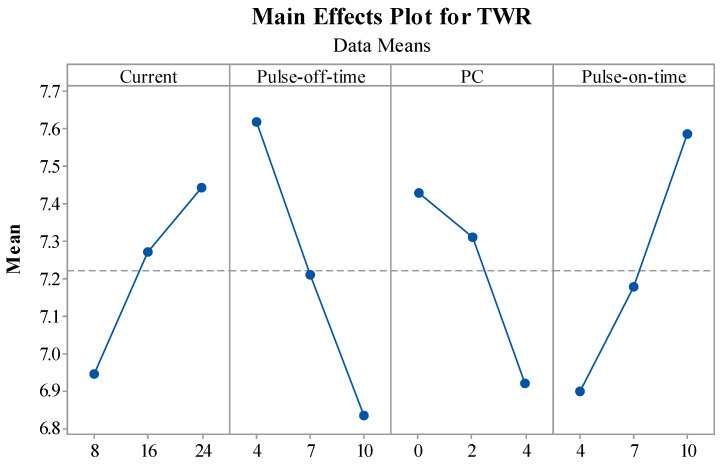
Impact of EDM variables on TWR.

Figure 6 shows the impact of the EDM variables on the output measures of TWR. EDM variables were represented on the X-axis, and the means of the TWR measure for the respective input were depicted on Y-axis. The main effect plot for current vs. TWR depicted a rise in the TWR owing to the escalation in the current values. The reason behind this rise in TWR was the higher input energy. As quick tool wear was noticed with an increase in current, it was discovered that the tools were more sensitive to the discharge current [48]. In the case of the increase in T_off_, TWR decreased, as the discharge energy was lowered due to the absence of a spark for a longer duration [49]. In the PC vs. TWR plot, it can be observed that, with the increase in the PC, the TWR decreased. Figure 6 demonstrates that a higher percentage of PC lowers the TWR in the case of the concentration of the powder. As a result, TWR was reduced. This is due to the bridging phenomena, which results in arcs and cuts down on pure machining time [20,46]. For the T_on_ vs. TWR plot, an increase in T_on_ resulted in higher TWR values. The reason behind this was the increased spark intensity and discharge energy [48]. The ideal levels for obtaining lower TWR include setting current and T_on_ at lower levels and PC and T_off_ at higher levels.

**Figure 7:**
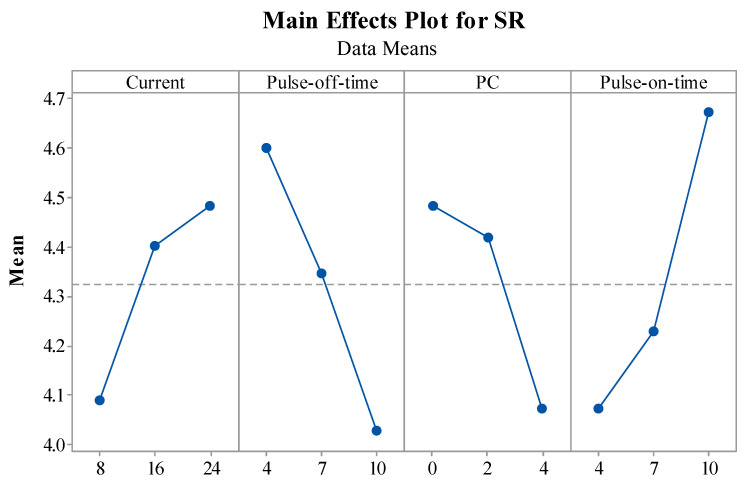
Impact of EDM variables on SR.

Figure 7 shows the impact of EDM variables on the output measures of SR. EDM variables were represented on the X-axis, and the means of the SR measure for each respective input were depicted on the Y-axis. In the main effect plot for current vs. SR, it was found that, as the current keeps increasing, the SR also increases. An increase in current causes an increase in discharge energy, increasing thermal energy. This thermal energy melts and vaporizes the work material. Higher temperatures are produced by these increases in thermal energy and sparks, which escalates the SR. Large and deep craters were formed on the work material due to an increased rate of erosion caused by an increase in current. This, in turn, produces a more even surface. A decrease in the value of SR was observed with an increase in the value of T_off_. It was found that increasing T_off_ had a positive impact on SR. Due to a rise in T_off_, a reduction in spark intensity further lowers the temperature. The surge in T_off_ lowers the thermal and discharge energy [50]. This resulted in a small crater and improved the smoothness of the surface of the material being machined. As a result, SR decreases when T_off_ increases. From the PC vs. SR plot, it can be observed that the SR decreases as the PC increases. By creating tiny craters and lowering the plasma heat flux, the addition of PC widens the interelectrode gap and boosts heat dissipation in the dielectric fluid. The use of nano-powder improved the flushing of waste from the machining zone [24]. Enhancing debris flushing created small ridges, improving the surface quality. For T_on_ vs. MRR, it was observed that, with an increase in the value of T_on_, the value of SR also increases. As the interval between pulse occurrences shrinks, more sparks occur among the tool and workpiece. More material is removed as the temperature rises and the spark generation increases. Large and deep craters formed on the work material as a result of an increased rate of erosion and T_on_ [50]. The machining surface becomes rougher and depreciates as a result. Thus, as T_on_ increases, SR does too. Ideal levels for obtaining a lower SR include setting current and T_on_ at lower levels and PC and T_off_ at higher levels.

### 3.6. Optimization

The main effect plots for the selected output measures have shown distinct levels of EDM variables for acquiring their desired performances, such as higher MRR and lower TWR and SR. Thus, finding an optimal parametric setting for attaining the desired levels of all the performance measures is essential. Pursuant to this, the TLBO technique has been implemented in the current study. Extreme limits of EDM variables, as mentioned in Table 1, were taken as upper and lower bounds while performing the optimization. Another necessary condition of positive integers has also been applied during the execution. The simultaneous optimization of all the performance measures was carried out by considering the equal weightage of MRR, TWR, and SR, i.e., 0.33 each. Pursuant to the present condition, the optimal parametric settings of current at 24 A, PC at 4 g/L, T_off_ at 10 µs, and T_on_ of 4 µs produced the optimal input parameters of 43.57 mg/min for MRR, 6.478 mg/min for TWR, and 3.73 µm for SR. These results obtained from the TLBO technique were validated by performing the experiments at the optimal parametric settings of the EDM process. Table 4 summarizes the predicted and actual results of the optimal parametric settings. The experimentally measured values were an MRR of 444.13 mg/min, a TWR of 6.331 mg/min, and an SR of 3.61 µm. Thus, these results from the experimental trial revealed a negligible variation of less than 5% in all performance measure outputs. Thus, the derived results revealed the acceptability and adequacy of the generated models and TLBO technique.

**Figure 8:**
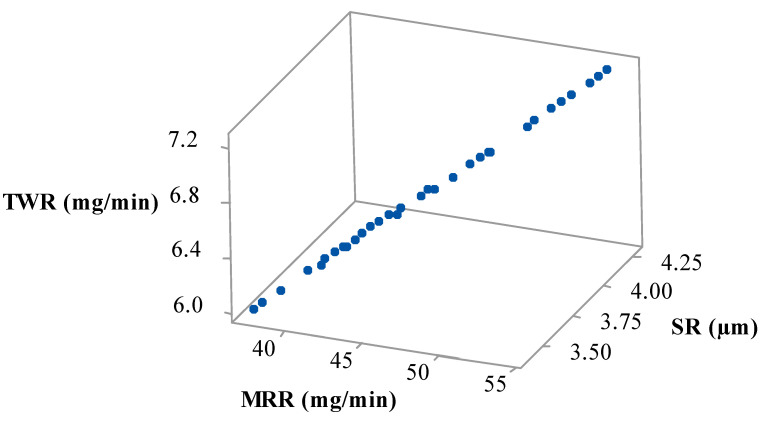
3D Pareto curve.

By considering the different user and application requirements, it is necessary to derive a set of optimal parameters. Pursuant to this, 40 Pareto points were generated, which contain all the unique solutions. Figure 8 depicts the Pareto curve for these 40 unique points. With the help of these multiple optimal points, users can select their preferred parametric settings by analyzing their requirements of output measures.

### 3.7. Surface Morphology for Nano-Powder

**Figure 9:**
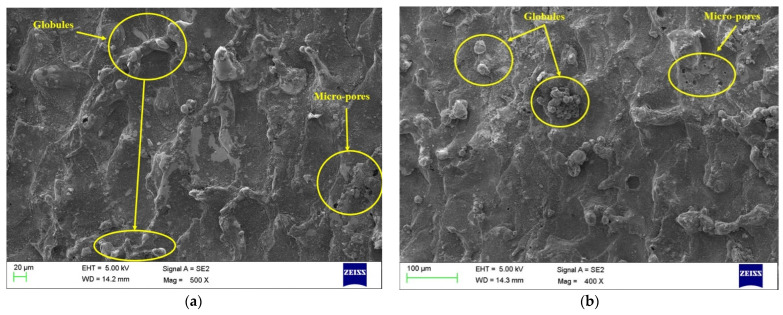
Machined surfaces for conventional EDM (without PC) for (**a**) top zone, (**b**) bottom zone.

**Figure 10:**
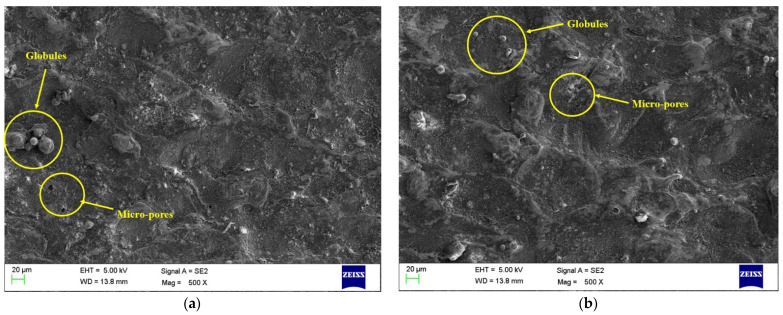
Machined surface for PMEDM (with Al_2_O_3_ PC of 4 g/L) for (**a**) top zone, (**b**) bottom zone.

The results from the main effect plots clearly show the positive impact of the addition of PC on all the performance measures. The addition of PC has shown an enhancement in MRR, TWR, and SR. Moreover, ANOVA results have found that PC had a significant variable for all the performance measures and was the highest contributor in the case of MRR. Thus, it becomes essential to study PC’s impact on the machined surface’s surface morphology through SEM. Pursuant to this, levels of optimized variables were considered for current, T_on_, and T_off_ to investigate the surface features with PC (4 g/L) and without PC (0 g/L). Figure 9 and Figure 10 depict SEM micrographs for the surface features of conventional EDM (0 g/L) and PMEDM (4 g/L) processes, respectively. SEM images were captured at two different locations for each sample to reproduce the accurate results. The SEM micrographs from Figure 9a,b of a conventional EDM process can be seen to have more surface deficiencies, such as the deposition of debris, micro-pores, globules, etc. On the other side, the surface features of the PMEDM process, as shown in Figure 10a,b, has a negligible amount of debris depositions, micro-pores, and globules. In addition to this, experimental values of the PMEDM process and conventional EDM process show SR values of 3.73 µm and 4.94 µm, respectively. This has shown an improvement of 32.43% in SR while using the PMEDM process with Al_2_O_3_ nano-powder at a concentration of 4 g/L. A uniform spark distribution between Nitinol and the tool ensures a reduction in the number of globules [51,52]. By creating tiny craters and lowering the plasma heat flux, the addition of PC widened the interelectrode gap and boosted heat dissipation in the dielectric fluid [53,54]. Use of PC has improved the flushing of waste from the machining zone [55]. Enhancing debris flushing created small ridges, improving the surface quality [56]. Thus, all these phenomena have resulted in an extensive drop in surface flaws. Thus, the obtained SEM graph established that an Al_2_O_3_ nano-powder concentration of 4 g/L improved the quality of the machined parts by decreasing the surface defects.

## 4. Conclusions

In the present study, the effect of alumina (Al_2_O_3_) nano-powder was investigated for the electrical discharge machining (EDM) of Nitinol shape memory alloy (SMA). The conclusions of the study were drawn as follows:

- ANOVA results showed that regression model terms significantly impacted the developed model terms in the case of all the performance measures. In the case of individual variables, PC, T_off_, and T_on_ significantly impacted the output measures of MRR, TWR, and SR, respectively. Verifying the normal probability plot yielded good ANOVA results and satisfied the necessary condition for ANOVA. Thus, all the statistical findings revealed the acceptability and fitness of the suggested models;
- The main effect plots were observed to have a positive impact on the addition of PC on all the performance measures. The addition of PC enhanced MRR, TWR, and SR;
- The TLBO algorithm has shown optimal parametric settings of current at 24 A, PC at 4 g/L, T_off_ at 10 µs, and T_on_ of 4 µs. Moreover, it has shown optimal input parameters of 43.57 mg/min for MRR, 6.478 mg/min for TWR, and 3.73 µm for SR. Pareto points with unique solutions were generated by considering the need of users for specific applications;
- Lastly, scanning electron microscopy (SEM) was used for the machined surface analysis. The obtained SEM graph established that using an Al_2_O_3_ nano-powder concentration of 4 g/L improved the quality of the machined parts by decreasing surface defects.

## Figures and Tables

**Table 1:** Die-sinking EDM parameters with actual values.

Parameters	Values
Current (A)	8; 16; 24
Pulse-off time (µs)	4; 7; 10
PC (g/L)	0, 2, 4
Pulse-on time (µs)	4; 7; 10
Size of nano-powder (nm)	100–150
Spark gap (mm)	0.01
Cutting depth (mm)	2
Tool	Copper

**Table 2:** Experimental plan and obtained results of MRR, TWR, and SR.

Trial	Current (A)	T_off_ (µs)	PC (g/L)	T_on_ (µs)	MRR (mg/min)	TWR (mg/min)	SR (µm)
1	8	4	0	4	22.87 ± 0.17	7.23 ± 0.17	4.27 ± 0.17
2	8	7	2	7	31.21 ± 0.31	6.98 ± 0.11	4.11 ± 0.11
3	8	10	4	10	36.71 ± 0.28	6.63 ± 0.22	3.89 ± 0.16
4	16	4	2	10	43.53 ± 0.22	8.13 ± 0.24	5.12 ± 0.22
5	16	7	4	4	47.04 ± 0.25	6.64 ± 0.13	3.92 ± 0.12
6	16	10	0	7	19.23 ± 0.11	7.05 ± 0.17	4.17 ± 0.13
7	24	4	4	7	53.18 ± 0.26	7.50 ± 0.19	4.41 ± 0.16
8	24	7	0	10	24.79 ± 0.18	8.01 ± 0.14	5.01 ± 0.19
9	24	10	2	4	31.98 ± 0.19	6.83 ± 0.16	4.03 ± 0.15

**Table 3:** ANOVA for MRR, TWR, and SR.

*Source*	*DF*	*SS*	*MS*	*F-Value*	*p-Value*
**MRR**					
*Regression*	4	1047.44	261.86	41.82	0.002
*Current*	1	61.24	61.24	9.78	0.035
*T_off_*	1	167.15	167.15	26.69	0.007
*PC*	1	817.39	817.39	130.53	0.000
*T_on_*	1	1.65	1.65	0.26	0.635
*Error*	4	25.05	6.26		
*Total*	8	1072.49			
**TWR**					
*Regression*	4	2.4037	0.6009	41.11	0.002
*Current*	1	0.3742	0.3742	25.60	0.007
*T_off_*	1	0.9249	0.9249	63.27	0.001
*PC*	1	0.3894	0.3894	26.64	0.007
*T_on_*	1	0.7151	0.7151	48.92	0.002
*Error*	4	0.0584	0.0146		
*Total*	8	2.4622			
**SR**					
*Regression*	4	1.5116	0.3778	13.68	0.013
*Current*	1	0.2321	0.2321	8.40	0.044
*T_off_*	1	0.4873	0.4873	17.65	0.014
*PC*	1	0.2521	0.2521	9.13	0.039
*T_on_*	1	0.5400	0.5400	19.56	0.011
*Error*	5	0.1105	0.0276		
*Total*	8	1.6220			

**Table 4:** Summary of predicted and actual results at optimal parametric settings.

Condition/Response	MRR (mg/min)	TWR (mg/min)	SR (µm)
**Predicted from TLBO**	43.57	6.478	3.73
**Experimental values**	44.13	6.331	3.61
**% Error**	1.28	2.26	3.21

## Data Availability

Data presented in this study are available in this article.

## Nomenclature

ANOVA	Analysis of variance
DF	Degree of freedom
DOE	Design of Experiments
EDM	Electrical discharge machining
IEG	Inter-electrode gap
MRR	Material removal rate (mg/min)
NPMEDM	Nano-powder-mixed electrical discharge machining
PC	Powder concentration
PMEDM	Powder-mixed electrical discharge machining
SEM	Scanning electron microscope
SMA	Shape memory alloy
SMAs	Shape memory alloys
SR	Surface roughness (µm)
TEM	Transmission electron microscope
TLBO	Teaching–learning based optimization
TWR	Tool wear rate (mg/min)
T_on_	Pulse on time (µs)
T_off_	Pulse off time (µs)
T	Time in minutes
